# The Infirmary Question at Manchester

**Published:** 1897-01-09

**Authors:** 


### THE INSTITUTIONAL WORKSHOP.

### THE INFIRMARY QUESTION AT MANCHESTER.

The infirmary question, as it is called in Manchester, has reached so important a stage that it seems fitting that we should give an impartial review of the situation, and indicate the lines on which some form of compromise ought to be arrived at. For compromise it must be, owing to the many interests which are involved.

The problem is a very complicated one. First of all there are the sick, then there are the authorities, and with them the students, of the Owens College, and then there is the public opinion of Manchester, which protests with no uncertain voice against the proposal to build over the open space by which the present Royal Infirmary is surrounded.

The present infirmary provides 298 beds, and it is agreed on all hands that this is far from being a sufficient number. But even the present beds are but imperfectly accommodated in the existing buildings. These have been thoroughly condemned, notwithstanding the various attempts at improvement which have from time to time been made; so that, even if the necessity for extension entered less prominently than it does into the question, the governors could not long continue satisfied with the existing condition of affairs.

But even in so small a matter as putting their house in order, without entering on the question of enlargement at all, the authorities are hampered by the fact that, although the infirmary stands on a fairly large plot of ground, this land is the main open space in the centre of Manchester, and any attempt to build over it excites the strongest possible opposition. This open space around the Royal Infirmary is very dear to the Manchester people, and there can be no doubt that the poll which recently divided the trustees into such nearly equal camps had to do mainly with the preservation of this land as a permanent open space for the centre of the city.

With this feeling on the part of the Manchester people we have every sympathy, but we need hardly say that if the inhabitants want the land, which is now vested in the hands of trustees for the good of the sick, the people, through their representatives, the Corporation, should buy it. This is the first point to which we would draw attention; that in all equity the Corporation should buy from the infirmary authorities, at a proper price, all such land as may be required by the city for its own advantage, either from a sanitary or an æsthetic point of view.

If the Corporation would do this, the remainder of the site would remain available for a small central hospital, but if they refuse, the only plan will be to sell the land and let people build warehouses on it as they like. It is not fitted for a large Royal Infirmary. The openness of the site makes a small hospital possible, but the proposal to enlarge the accommodation of the infirmary, while at the same time destroying the openness of the site, seems to us a suicidal suggestion. The trustees, however, cannot afford to confine their efforts to the improvement of their present accommodation. Unless they provide more beds, the Royal Infirmary will be snuffed out by some new institution; for whatever hospital has the medical school, that will be the leading hospital of the city. The hands of the trustees are forced in this matter by the existence of a standing difficulty with the authorities of the Owens College, and it seems clear that no settlement of the infirmary question can be looked on as permanent which does not include a settlement of this perennial source of controversy. This college is a great and valuable institution which has done good service to Manchester and the surrounding districts, and of which the district is proud in no small degree; yet it is crippled in its medical department by its professors of medicine and surgery having no rights in the Royal Infirmary. The college already possesses a site for a new hospital under its own control, from the utilisation of which, however, it has hitherto refrained, led no doubt by the knowledge that the sympathies of the great givers lie with the old Royal Infirmary. But once let the infirmary trustees adopt a thoroughly unpopular course, as by building over the Piccadilly site; or let them confess that they cannot provide a suitable number of beds, and the waters will be loosed, the subscribing public will be split into factions, and the Owens College will at last get its wish and obtain beds of its own for clinical teaching, although only at the cost of being saddled with a white elephant in the form of a hospital. The trustees of the infirmary, then, must put forward a scheme offering beds enough, or the very thing will happen which they most dread—viz., that the new accommodation, which is bound to come, will come in the form of a new hospital attached to Owens College—and then the Royal Infirmary will lose the students, and sink into a second-rate position.

It is clear, then, that the question, as it appeals to the people of Manchester, is a complicated problem, and that it is not to be decided, and, in fact, is hardly likely to be discussed, on the simple basis of what is best for the sick. Whatever decision is arrived at must be of the nature of a compromise; but each party need not give way in equal proportion, and we think it is possible to keep steadily in view the interests of the sick, while still doing all that need be done both to obtain the loyal co-operation of the college, and to conciliate the sentiment of the town.

So far as economy of management and the good of the patients are concerned, it is most important that the trustees should remain the Infirmary Board for Manchester, and that there should not be separate competing infirmaries. The only real difficulty as to this would seem to arise from the present not unnatural unwillingness of the college authorities to co-operate in any scheme which does not give their professors of medicine and surgery the right to positions on the staff of the hospital. But in view of the very great advantage which the hospital gains from its association with the school, and the very awkward position in which the infirmary would find itself if deprived of its students—as it would be, to a large extent, if the college were once able to get a hospital even of a hundred and fifty beds for clinical purposes near its own gates—we think it is worth a very considerable sacrifice on the part of the infirmary authorities — a sacrifice far greater than is involved in the admission of the College Professors to their wards —for the sake of keeping in their own hands the administration of the enlarged infirmary, whether it be in one building or in several. We think, then, that it ought to be accepted as the basis for any action which may take place, whether a separate hospital be built or not, that the management should remain in the same hands, and that at all costs the hospital and the college should be firmly linked together both by interest and by law, so that while the college shall feel sure of always having a full field for teaching purposes, the hospital shall feel sure of always retaining for its patients the inestimable advantages of being attached to the Owens College. That being granted, we have next to consider whether it will be best, after disposing of that portion of the site required by the city as an open space, to put up a grand new hospital on a new site, or endeavour to crowd the required accommodation upon the smaller area remaining to them, or to erect on this area a first-rate emergency hospital, together with a well-equipped out-patient department, while removing the larger portion of the hospital to some other locality. It need hardly be said that the latter is the alternative which would most completely satisfy the public sentiment.

There is no doubt that economical considerations are in the abstract in favour of the single institution; but when it becomes a question of using so valuable a site as that on which the present building stands a good deal of the cogency of this consideration falls to the ground, while, on the other hand, if it became a question of building the hospital in some suburban quarter the managers would at once be faced with the difficulty of the out-patients, a central out-patient department would be a necessity, and we do not think it would be long before the managers would be driven to attach a few emergency wards to this department wherever it might be established. This is a point that must be thoroughly grasped—that it is out of the question to move the infirmary into the suburbs and leave a great city like Manchester without emergency beds. The question then of a single central *versus* a single suburban hospital really does not arise, and the proposal to have a single hospital resolves itsely of necessity into a proposal to have one in the centre of the city. So far as this goes the proposals of the Grand Committee are perfectly logical and reasonable; but it is to be borne in mind that the form these proposals take is in itself a strong argument against attempting to place the whole of the required accommodation on the present site, for if this can only be done at the expense of such a destruction of open space as is there detailed the proposal stands condemned from the townspeople's point of view, while, as a matter of economy, the necessity of using so large a proportion of so valuable a site merely for the sake of obtaining light and air makes it a somewhat questionable proceeding.

But, overshadowing every proposal to build a complete hospital on the present site, there is an aspect of the question which must not be lost sight of. In our opinion, the only possible excuse for such a proceeding is the advantage, which from an administrative point of view we admit is a great one, of having the whole institution together in one place. But one has only to look ahead, even a short distance, into the future, to feel very sure that Manchester will not be permanently content with the hospital accommodation which can be safely provided on the Piccadilly site, and thus, whenever the time arrives when more accommodation shall be required, the whole question will have to be reopened, and a second hospital will of necessity have to be erected. In view, then, of the fact that sooner or later a second hospital will have to be erected, we advise that it be erected now; that it be placed on the best site that can be found, due provision being made for future extensions; and that, after the corporation has purchased such of the present site as it requires for sanitary and æsthetic purposes, the rest be used for the erection of a very perfect emergency hospital of perhaps one hundred beds, together with an out-patient department provided with all the most recent appliances for the best clinical instruction in all the specialities.

We can have no possible doubt that this plan will be far the best for the sick; while, in regard to the school, there is no reason to anticipate any insurmountable difficulty. If a bald out-patient department, a mere dispensary, without beds, were established far away from the hospital, the students would have undoubted cause for complaint; but with a central hospital of, say, 100 beds, full of urgent cases at the one place, and with a great general hospital of, say, 400 beds at the other, no student need ever complain of want of opportunities.

This seems to us the proper solution of the difficulty. A certain loss must be sustained by some one if the land at present belonging to the infirmary is to be kept in perpetuity as an open space for the good of the town. In all justice this loss should fall on the town, and the land so required should be purchased by the corporation. Whatever happens there must be a central hospital both for the reception of accidents and for the full development of the utility of the out-patient department, both as regards the patients and the school; but, beyond the accommodation required for this purpose, all the rest of the beds should be removed to a more commodious and airy site, where such extensions may be made as may in the future be found necessary, and where such classification of the patients may be effected as the requirements of an advancing science may demand, and where especially such a permanent association with the college shall be secured that whenever in the future still further hospital accommodation may be required, it may be provided in harmony with, and not in opposition to, the Royal Infirmary, the trustees of which would thus become the central hospital authority for the great and growing city of Manchester.

